# The influence of exercise on clinical pain and pain mechanisms in patients with subacromial pain syndrome

**DOI:** 10.1002/ejp.2010

**Published:** 2022-07-27

**Authors:** Kristian Damgaard Lyng, Jonas Dahl Andersen, Steen Lund Jensen, Jens Lykkegaard Olesen, Lars Arendt‐Nielsen, Niels Kragh Madsen, Kristian Kjær Petersen

**Affiliations:** ^1^ Department of Health Science and Technology, Faculty of Medicine Aalborg University Aalborg Denmark; ^2^ Center for General Practice at Aalborg University, Department of Clinical Medicine Aalborg University Aalborg Denmark; ^3^ Department of Orthopaedic Surgery Shoulder Unit, Aalborg University Hospital, Farsø Hospital Aalborg Denmark; ^4^ Department of Clinical Medicine, Faculty of Medicine Aalborg University Aalborg Denmark; ^5^ Center for Neuroplasticity and Pain (CNAP), SMI, Department of Health Science and Technology, Faculty of Medicine Aalborg University Aalborg Denmark; ^6^ Department of Gastroenterology and Hepatology, Mech‐Sense Aalborg University Hospital Aalborg Denmark; ^7^ Center for Mathematical Modeling of Knee Osteoarthritis (MathKOA), Department of Material and Production, Faculty of Engineering and Science Aalborg University Aalborg Denmark

## Abstract

**Background:**

Few studies have investigated the underlying mechanisms for unilateral subacromial pain syndrome (SAPS). Therefore, this study examined (1) if 8‐weeks of exercise could modulate clinical pain or temporal summation of pain (TSP), conditioned pain modulation (CPM), and exercise‐induced hypoalgesia (EIH) and (2) if any of these parameters could predict the effect of 8‐weeks of exercise in patients with unilateral SAPS.

**Methods:**

Thirty‐seven patients completed a progressive abduction exercise program every other day for 8‐weeks. Worst shoulder pain in full abduction was rated on a numeric rating scale (NRS). Pain pressure thresholds (PPTs), TSP, CPM, EIH, Shoulder Pain and Disability Index (SPADI), Pain Catastrophizing Scale (PCS), PainDETECT questionnaire (PD‐Q), Pain Self‐Efficacy Questionnaire (PSE‐Q) and Pittsburgh Sleep Quality Index (PSQI) were assessed before and after intervention.

**Results:**

The intervention improved worst pain intensity (*p* < 0.001), increased the CPM (*p* < 0.001), improved the sleep scores (*p* < 0.005) and reduced the PainDETECT ratings (*p* < 0.001). No changes were observed in PPT, TSP, EIH, SPADI, PCS and PSE‐Q (all *p* > 0.05). In a linear regression, the combination of all baseline parameters predicted 23.2% variance in absolute change in pain after 8 weeks. Applying backwards elimination to the linear regression yielded that baseline pain intensity combined with TSP predicted 33.8% variance.

**Conclusion:**

This explorative study suggested reduction in pain, improved sleep quality and increased CPM after 8‐weeks of exercise. Furthermore, the results suggests that low pain intensity and high TSP scores (indicative for pain sensitisation) may predict a lack of pain improvement after exercise.

## 1 INTRODUCTION

Shoulder pain is among the most common form of musculoskeletal pain (Picavet & Schouten, 2003; James et al., 2018). It has been reported that 70% of shoulder pain involves the rotator cuff with the diagnosis of subacromial pain syndrome (SAPS) being the most frequent (van der Windt et al., 1996; Juel & Natvig, 2014; Bhattacharyya et al., 2014). SAPS is often characterized as localized pain and swelling seen in the affected tendon, minimal resting pain, almost normal range of motion and pain through resistance training and activity (Andres & Murrell, 2008; Couppé et al., 2015; Millar et al., 2021; Hermans et al., 2013). The underlying mechanisms for shoulder pain and SAPS have currently, not been fully established (Dean et al., 2013). Quantitative sensory testing (QST) has been specifically developed also for assessment and profiling of musculoskeletal pain conditions (Graven‐Nielsen & Arendt‐Nielsen, 2010; Arendt‐Nielsen et al., 2018) and may involve pressure pain thresholds (PPTs), temporal summation of pain (TSP), conditioned pain modulation (CPM) and exercise‐induced hypoalgesia (EIH) (Dyck et al., 1993; Rolke et al., 2006; Petersen et al., 2019a; Petersen et al., 2016; Hansen et al., 2020a). Shoulder pain has previously been associated with widespread pressure hyperalgesia (Paul et al., 2012). Preliminary evidence suggests that shoulder pain may cause facilitated TSP and impaired CPM (Valencia et al., 2012), which are common findings for other musculoskeletal pain conditions (Arendt‐Nielsen et al., 2018) suggesting sensitization of central pain pathways. Emerging evidence suggests that QST might hold predictive value for the outcomes of specific pain therapies such as exercise, pharmacological treatments, and surgery (Arendt‐Nielsen et al., 2018; Petersen et al., 2015; Petersen et al., 2020; Lyng et al., 2021; Yarnitsky et al., 2012) which may also adhere to shoulder pain (Lyng et al., 2021). Studies in patients with neck/shoulder pain have suggested that CPM and TSP can be modulated (Yarnitsky et al., 2012; GravenNielsen et al., 2000) by exercise and that 5‐weeks exercise therapy may increase CPM (Heredia‐Rizo et al., 2018). Despite the fact that exercise is considered as first‐line of care in SAPS (Doiron‐Cadrin et al., 2020) no studies have investigated the modulatory effects of exercise on a specific set of QST parameters and their possible predictive value for outcome.

This study aimed to examine (1) if 8‐weeks of exercise could modulate the clinical pain and QST and (2) if any of the parameters could predict the effect of exercise in patients with unilateral SAPS.

## 2 METHODS

### 2.1 Participants

**FIGURE 1:**
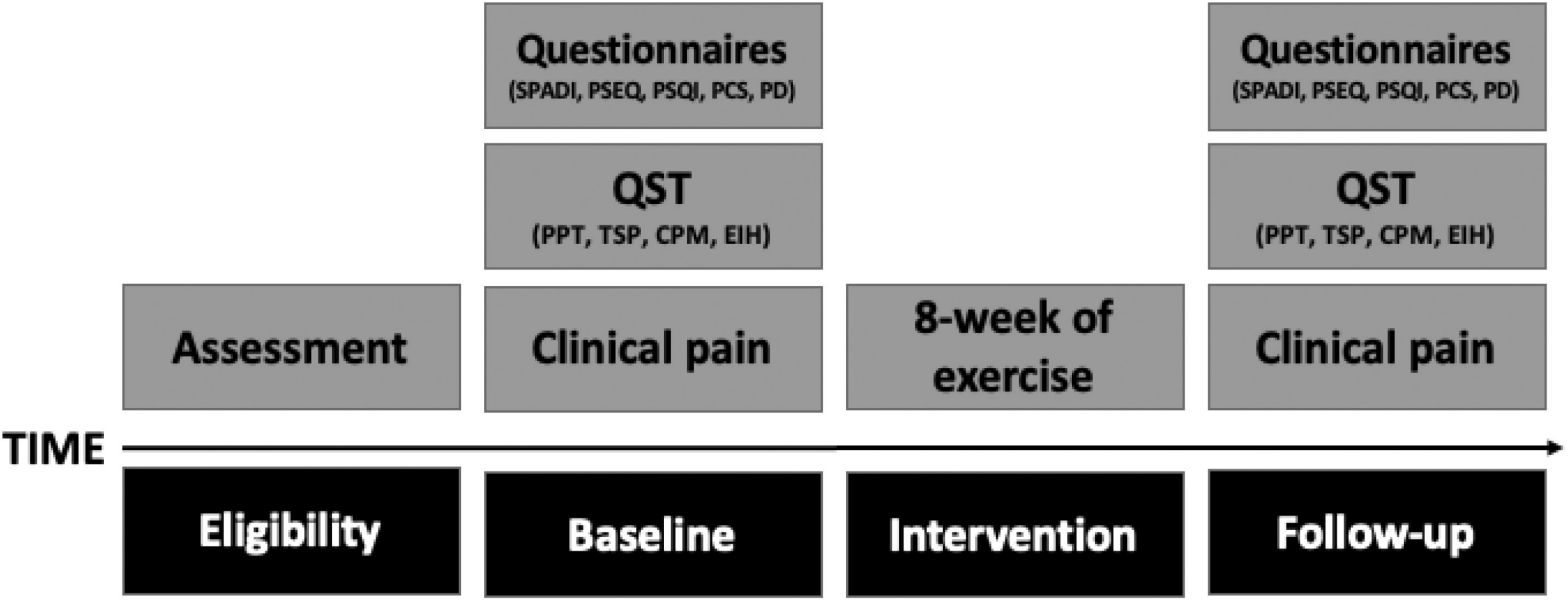
Experimental procedure. CPM, Conditioned Pain Modulation; EIH, Exercise‐induced Hypoalgesia; PCS, The Pain Catastrophizing Scale; PD, PainDETECT; PPT, Pressure Pain Threshold; PSEQ, Pain Self‐Efficacy Questionnaire; PSQI, Pittsburgh Sleep Quality Index; SPADI, Shoulder Pain and Disability Index; TSP, Temporal Summation of Pain. Assessment refers to the clinical screening (including ultrasound of the shoulder joint) for study eligibility.

A consecutive cohort of participants aged 18–65 and diagnosed with unilateral SAPS were recruited from the Department of Orthopaedic Surgery, Aalborg University Hospital, Farsø or RheumaNord, Aalborg or via social media (i.e., Facebook). Inclusion criteria were persistent pain at the proximal lateral aspect of the upper arm for ≥3 months (Numeric Rating Scale [NRS, 0–10] ≥3); at least 3 positive findings on a 7‐item test‐cluster of pain in abduction, shrug‐sign, full‐can, Jobe, Hawkins‐Kennedy, Neer test and a resisted external rotation (Park et al., 2005); minimal to no limitation of passive shoulder range of movement; and ultrasound evidence of structural abnormalities (e.g., hypoechoic areas, fibrillar disruption, neovascularization, calcifications embedded in tendon and oedema) (Ingwersen et al., 2016; McCreesh et al., 2016). Exclusion criteria included the presence of other shoulder problems (e.g., rotator cuff tear); neurological diseases; previous shoulder surgery; pregnancy; and pain medication use 24 h prior to testing; corticosteroid injection in the affected shoulder joint in the past 6 months; substance abuse; and mental impairment. This assessment was performed prior to data collection (See Figure 1). Eligible participants were asked to refrain from other concurrent treatment while enrolled in the study and on test days. This study was approved by The North Denmark Region Committee on Health Research Ethics (N‐20190046) and conducted in accordance with the Helsinki Declaration. Written informed consents were obtained. Data were collected between October 2019 and April 2021 by the same tester (KDL). Sample sized was estimated using Gpower 3.1.9.4 (Kiel University), to detect a clinically relevant reduction in pain intensity after 8‐weeks of intervention. To account for dropout and to detect an estimated reduction in clinical pain larger than 2 points on NRS was assumed after the intervention in comparison with baseline measurements. Therefore, we computed a sample size of 37 in total to test the hypothesis with 80% power and a significance level of *p* < 0.05.

### 2.2 Procedure

This exploratory study consisted of two sessions of baseline and 8‐weeks follow‐up visits (a seven‐day buffer for follow‐up visit was accepted). Each 2‐h session consisted of a test battery of quantitative sensory tests, questionnaires, and an ultrasound scan. Baseline demographic data (sex, age, height, weight and prior or concurrent use of pain medication and if yes, which type) were recorded. After the baseline session, participants were instructed in performing a simple shoulder exercise daily for the following 6 weeks. All participants were familiarized with every procedure as standardized instruction was issued for all participants. See Figure 1 for full experimental procedure.

#### 2.2.1 Clinical pain

Worst perceived pain intensity on full shoulder abduction, average pain intensity reported within the last 24 h and duration (months) of pain was recorded at both sessions. Worst perceived pain intensity was used as primary outcome for the study, as this is commonly used in studies on QST (Petersen et al., 2016; Petersen et al., 2015; Petersen et al., 2019b; Petersen et al., 2019c). Minimal clinically important difference (MCID) is the term used for describing the smallest change in outcome that patients perceive as meaningful (Cook, 2013). Multiple studies within different musculoskeletal pain conditions are suggestive of at least 2 points decreased in NRS 0–10 to be counted as a MCID for worst perceived pain (Michener et al., 2011; Werner et al., 2016; Childs et al., 2005).

### 2.3 Experimental procedures

#### 2.3.1 Pressure pain threshold

A handheld pressure algometer (Somedic Sales AB) with a stimulation area of 1 cm^2^ was used to assess PPT. Pressure was applied with a probe perpendicular to the skin, the applied force was increased by 30 kPa/s. All measures were assessed in a sitting position and participants were instructed in pressing a stop‐button when the first onset of pain was experienced. To investigate local pain sensitivity, PPTs were assessed on the supraspinatus muscle (middle point over the fossa of the scapula), distally PPTs were also assessed on the extensor carpi radialis brevis (ECRB) (muscle belly) and remotely on m. quadriceps (the middle of the dominant quadriceps muscle, 15 centimetres proximal from the basis of patella). Three measurements were conducted at each site and an average was used for further analysis. All measurements were taken bilaterally. The measurements at all sites were repeated immediately after a single bout of exercise to determine EIH.

#### 2.3.2 Pinprick induced temporal summation of pain

TSP induced by pinprick stimulus was assessed using a von Frey stimulator with a weighted load (Aalborg University). The participants rated the pain intensity on the NRS (0–10), while the weighted load applied 25.6 g of force on their affected shoulder. Ten stimuli were applied (1‐s interval between stimulations) to the most painful site and the participants rated the pain intensity to the first and last stimuli. TSP was calculated as the difference in pain intensity between the first and the last stimulation (Heredia‐Rizo et al., 2018).

#### 2.3.3 Cuff algometry

The evaluation of deep‐tissue pain sensitivity was assessed by cuff pressure stimuli using a computer‐controlled cuff algometer (Cortex Technology, Hadsund and Aalborg University). The algometer includes two 13‐cm wide tourniquet cuffs (VBM) and an electronic VAS (Aalborg University) enabling continuous measurement of pain intensity. The cuff was placed at the level of the muscle belly of the gastrocnemius muscle at the ipsilateral site of the most painful shoulder. The electronic continuous VAS (sliding resistor) was 10 cm and was sampled at 10 Hz; 0 cm indicating “no pain” and 10 cm indicating “maximum pain”.

#### 2.3.4 Pain detection and tolerance

The computer‐controlled cuff algometer was used to determine the moment when the participants pain during the cuff algometry exceeded 1 cm on a VAS‐score, defined as pressure pain detection threshold (cPDT). The participants were instructed to press a stop‐button when their pain tolerance level was reached and was defined as their pressure pain tolerance threshold (cPTT). The pressure during the cuff algometry was increased by 1 kPa/s and the participants were instructed to rate the pain intensity continuously on the electronic VAS. cPDT and cPTT was assessed on both legs.

#### 2.3.5 Cuff algometry induced temporal summation of pain

TSP assessed using computer‐controlled cuff algometer was done with 10 short‐lasting stimuli (1 s each) at the level of the cPTT. One second break was used between each stimulus and meanwhile the participants were instructed to continuously rate the sequential stimuli using a VAS score to rate their pain intensity. Participants were instructed not to return the slider to zero during the 1 s breaks. TSP using the cuff algometry was defined as the difference between the tenth and the first VAS score, which was extracted for each.

#### 2.3.6 Conditioned pain modulation (CPM)

Cuff algometry was used to investigate CPM, which was assessed by the changes in cuff pain detection threshold (cPDT) sensitivity with and without a conditioning stimulus. A constant pressure stimulus induced by cuff was applied to the contralateral leg acting as the conditioning stimulus. The pressure was determined from the pressure equivalent to 70% of the participants pain tolerance threshold (cPTT) level as per previous studies (Graven‐Nielsen et al., 2017; Imai et al., 2016). The CPM‐effect was calculated as the difference in cPDT before and during the conditioned stimulus.

#### 2.3.7 Exercise‐induced hypoalgesia (EIH)

All subjects were asked to perform an isometric wall‐squat exercise for a maximum duration of 3 min or until fatigue. Participants were asked to stand against a wall with a shoulder width stance, both hands by the side and 100° of flexion in their knees (a goniometer placed along the femur was used to ensure the right angle). Participants were instructed to rate their pain (NRS) and fatigue (BORG CR10, 0 = no effort and 10 = maximal effort) in the legs after 0, 1, 2 and 3 min. This has previously been utilized to evoke EIH in healthy and chronic patients (McCreesh et al., 2016; Petersen et al., 2019b).

### 2.4 Questionnaire data

#### 2.4.1 Shoulder pain and disability index

The Shoulder Pain and Disability Index (SPADI) is a 13‐item form measuring function (five items) and pain (eight items) (Roach et al., 1991). The items were rated based on a scale from 0–10 (none—worst). The two groups (function and pain) are summed separately and weighted equally. Each subscale (function and pain) was treated separately as accordingly to a recent Rasch analysis of the validated Danish cross‐cultural adaptation of SPADI (Christiansen et al., 2012; Christensen et al., 2019).

#### 2.4.2 Pain catastrophizing scale

The Pain Catastrophizing Scale (PCS) is a 13‐item self‐report measure of pain‐related catastrophizing, and it assesses rumination, magnification, and helplessness (Sullivan et al., 1995). The participants were required to score previously painful experiences on a Likert scale ranging from 0 = not at all to 4 = all the time.

#### 2.4.3 PainDETECT

The PainDETECT questionnaire is a self‐reported questionnaire, which contains three 11‐point numerical rating scales (Freynhagen et al., 2006). The participants were asked to answer different questions concerning highest and average pain intensity, pain quality and spatial distribution during the past four weeks. The pain intensity items are not included in the calculation of the final score of PD‐Q. PD‐Q uses a validated algorithm to calculate a PainDETECT score. The minimal score is 0 and maximal score is 38 and scores above 18 suggest neuropathic pain involvement (Karasugi et al., 2016; Jespersen et al., 2010). A validated Danish version was used (Kjøgx et al., 2014). A reduction in score was considered an improvement.

#### 2.4.4 Pain self‐efficacy questionnaire

The Pain Self‐Efficacy Questionnaire (PSEQ) is a 10‐item self‐report measure of self‐efficacy beliefs in people with chronic pain (Nicholas, 2007). The questions are related to a variety of different activities and the participants are to rate how confident they are in doing these tasks. Each activity is rated on a 7‐point scale, where 0 = not at all confident to 6 = completely confident. A validated Danish version was used (Rasmussen et al., 2016).

#### 2.4.5 Pittsburgh sleep quality index

The Pittsburgh Sleep Quality Index (PSQI) is a self‐rated questionnaire which assesses sleep quality and disturbances over a one‐month period (Buysse et al., 1989). 19 individual items generate seven “component” scores: subjective sleep quality, sleep latency, sleep duration, habitual sleep efficiency, sleep disturbances, use of sleep medications and daytime dysfunction. The sum of scores for these seven components yields one global score, which was utilized in this project. Higher scores are indicative of worse sleep quality.

#### 2.4.6 Intervention

Participants were instructed to perform a shoulder scaption exercise with the affected shoulder as seen in other studies (Bateman & Adams, 2014; Holmgren et al., 2014). The exercise involved lifting the arm into scaption against a therapeutic elastic band for three sets of 15 repetitions twice per every other day. Participants were told that pain during exercise was allowed if tolerable and if the pain were steadily reduced immediately after exercise (Smith et al., 2017). If the participants experienced absence of pain during the exercise and simultaneously were able to perform more than 15 repetitions per set, they were asked to progress to a band with more resistance. All participants were equipped with five different levels of band resistance. Participants logged their training throughout the study (reps, sets and sessions completed). Pain during exercise was noted using NRS 0–10 for each session. Adherence were calculated as the number of total sessions completed out of 64 sessions (2 sessions a day, 4 times per week for 8 weeks). See Appendix S1 for full description of intervention (Template for Intervention Description and Replication [TIDieR] checklist) (Hoffmann et al., 2014).

### 2.5 Statistical analysis

If not otherwise stated, all data are presented as mean, standard deviation (SD) and 95% confidence interval (CI). Non‐normality distributed data are expressed as medians with the 25th–75th interquartile range (IQR_25_, IQR_75_). Data were checked for normality using Shapiro–Wilk test (*p* > 0.05) and if normally distributed, parametric statistics were used and if not, non‐parametric tests were used. Changes from baseline to 8‐weeks follow‐up (i.e., the modulatory ability) in clinical pain, QST and questionnaire data were compared using paired sample test (*t*‐test or Wilcoxon). Bonferroni corrections were applied when needed to control for multiple comparisons. Multiple linear regression analyses using baseline parameters used to predict the effect of the intervention (absolute change in pain intensity comparing baseline and follow‐up scores) as the dependent parameter. Backwards selection was applied to the linear regressions to identify independent predictors using cut‐offs for statistical independence and inclusion of 0.05 and exclusion of 0.157, respectively, according to Akaike's Information Criterion for prognostic models (Heinze & Dunkler, 2017). All statistical analyses were computed using Statistical Package for Social Sciences (version 25; IBM Corporation). A *p*‐value of <0.05 will be considered statistically significant.

## 3 RESULTS

### 3.1 Demographics

**FIGURE 2:**
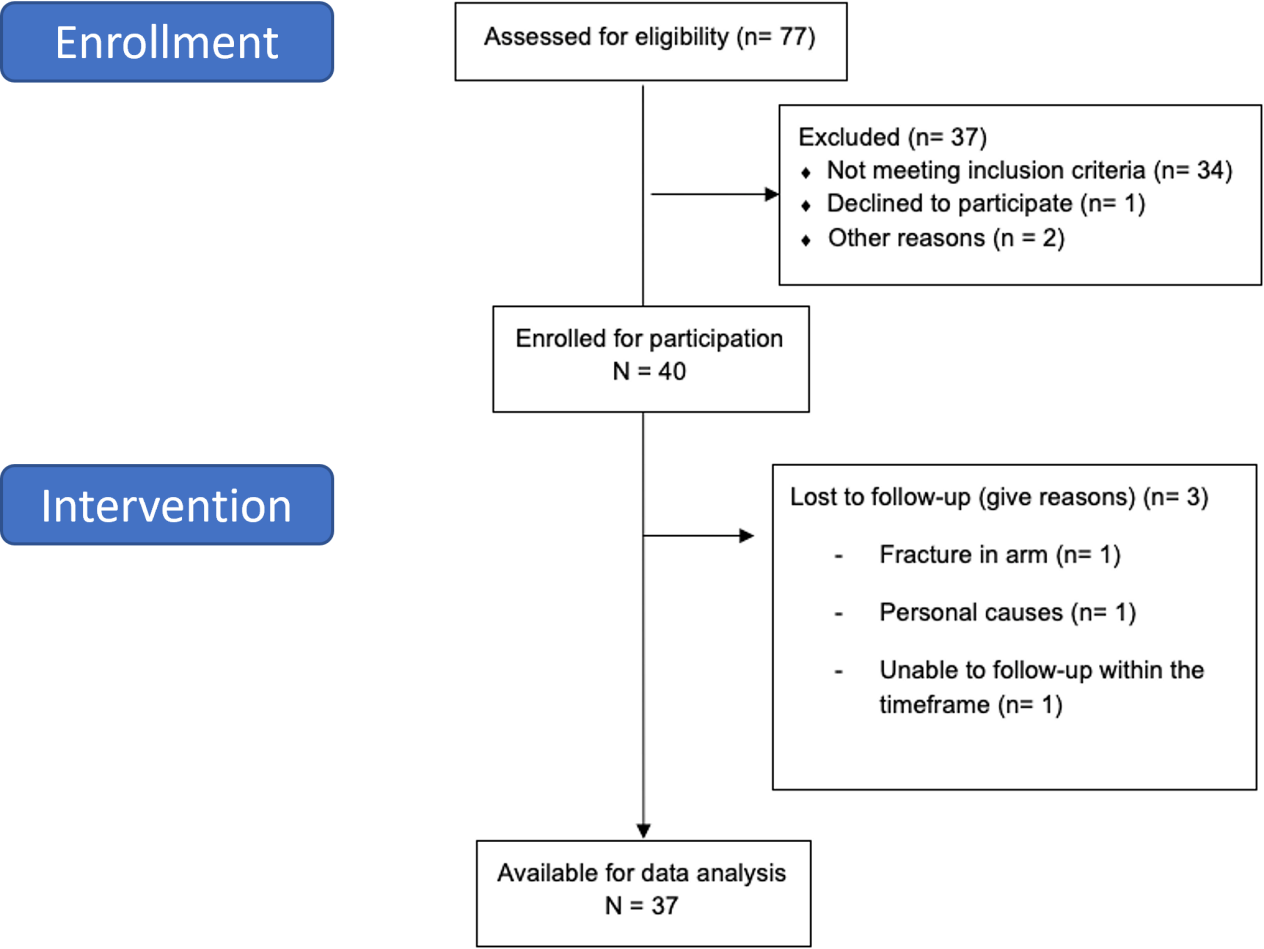
CONSORT flowchart

Of 77 participants screened, 40 were diagnosed with unilateral SAPS and eligible for this study. Three participants were unable to complete the study (1 fracture caused by fall, 1 personal causes, 1 failed to show up for follow‐up), leaving a sample of 37 for full analysis (see Figure 2). Demographics, clinical pain characteristics and questionnaire‐scores at baseline and follow‐up are summarized in Table 1. Based on self‐reported information from training diary, adherence was calculated to 78.5% (range 40–100). The average self‐reported pain while performing the exercise was 5.1 ± 3.5. 81% had previously used over‐the‐counter pain medication (i.e., Paracetamol, Ibuprofen) and 32% opioids (e.g., Morphine, Tramadol) to manage their pain.

**TABLE 1:** Demographics inclusion

Number (*n*)	37
Age, years	42 ± 9.5
BMI kg/m^2^	26.2 ± 4.6
Sex (*n* = ♀, *n* = ♂)	(*n* = 24, *n* = 13)
Duration of symptoms (months)	42.5 ± 42.1

*Note*: Baseline demographics. Results are presented as mean ± SD.

### 3.2 Effect of the intervention

The 8‐weeks intervention significantly decreased worst pain intensity within the last 24 h from 7.3 ± 1.4 to 5.7 ± 2.0 (mean change 1.5 ± 2.5, 95% CI [0.6, 2.4], *p* < 0.001). Average pain intensity reported within the last 24 h also improved from 4.5 ± 1.6 to 3.5 ± 1.9 (mean change 1.1 ± 2.0, 95% CI [0.3, 0.4], *p* < 0.005). The intervention significantly reduced PSQI score (from 9.3 ± 4.4 to 7.2 ± 4.4, mean change 2.1 ± 4.4, 95% CI [0.7, 3.6], *p* < 0.005), lowered PainDETECT score (from 9.5 ± 6.5 to 4.3 ± 4.1, mean change 5.0 ± 5.1, 95% CI [3.3, 6.7], *p* < 0.001). No change was observed in either subscale of SPADI, PSEQ, and PCS (*p* > 0.05).

### 3.3 Quantitative sensory testing outcomes

The QST parameters pre‐ and post‐intervention, and changes between sessions are listed in Table 2.

**TABLE 2:** Pre‐exercise vs post‐intervention differences

Variable	Pre‐exercise	Post‐exercise	Difference	*p* ^a^
Highest pain last week (NRS)	7 ± 1.3	6.2 ± 2.2	1.5 ± 2.5, CI (0.6, 2.4)	**<0.001**
Average pain (NRS)	4.5 ± 1.6	3.4 ± 2.2	1.1 ± 2.1, CI (0.4, 1.8)	**<0.001**
SPADI	44.08 ± 20.4	50.19 ± 24.29	−6.1 ± 26.3, CI (−14.8, 2.6)	0.16
painDETECT	9.5 ± 6.5	4.3 ± 4.1	5.0 ± 5.1, CI (3.3, 6.7)	**<0.001**
PSEQ	46 ± 12	46.9 ± 13.5	0.3 ± 14.1, CI (−4.3, 5.0)	0.88
PCS	18.7 ± 12.5	18.7 ± 13.7	0.08 ± 14.8, CI (−4.8, 5.0)	0.97
PSQI	9.3 ± 4.4	7.2 ± 4.4	2.1 ± 4.4, CI (0.7, 3.6)	**<0.005**
PPT Supraspinatus^b^ (kPa)	381 ± 179	365 ± 182	16.1 ± 171, CI (−41.1, 73.3)	0.57
PPT Elbow^b^ (kPa)	277 ± 128	252 ± 112	24.9 ± 134, CI (−19.8, 69.8)	0.26
PPT Quadriceps^b^ (kPa)	684 ± 238	689 ± 209	−5.0 ± 238.5, CI (−84.6, 74.4)	0.89
TSPpinprick (VAS)	2.5 ± 2	2.2 ± 1.6	0.2 ± 2.1, CI (−0.4, 1.0)	0.45
TSPcuff (VAS)	2.8 ± 1.6	3.0 ± 2.2	−0.2 ± 2.7, CI (−1.1, 0.7)	0.64
cPDT (kPa)	24 ± 10.2	24.4 ± 14.9	0.3 ± 2.8, CI (0.6, 5.4)	0.44
CPM (kPa)	29 ± 18.9	38.6 ± 19.4	9.5 ± 16.9, CI (3.9, 15.2)	**<0.001**
EIH_supraspinatus_ (kPa)	6.5 ± 91	36 ± 92	−29.4 ± 101, CI (−63.1, 4.2)	0.84
EIH_elbow_ (kPa)	3.8 ± 74	23 ± 78	−19.3 ± 101.6, CI (−53.2, 14.5)	0.25
EIH_quadriceps_ (kPa)	20.8 ± 153	48.3 ± 181	−27 ± 225.2, CI (−102.6, 47)	0.46

Abbreviations: CPM, Conditioned Pain Modulation; EIH, Exercise‐induced Hypoalgesia; kPa, kilo pascal; NRS, Numeric Rating Scale; PCS, The Pain Catastrophizing Scale; PD, PainDETECT; PPT, Pressure Pain Threshold; PSEQ, Pain Self‐Efficacy Questionnaire; PSQI, Pittsburgh Sleep Quality Index; SPADI, Shoulder Pain and Disability Index; TSP, Temporal Summation of Pain; VAS, Visual Analogue Scale. All values shown in mean ± standard deviation (SD). Statistical significant test highlighted in bold font. CI = 95% Confidence Interval. A Bonferroni correction was applied to account for multiple comparisons.

^a^ Paired‐samples t tests.

^b^ PPT only reported from pre‐wall‐squat test.

#### 3.3.1 Cuff pressure pain threshold and exercise‐induced hypoalgesia

**FIGURE 3:**
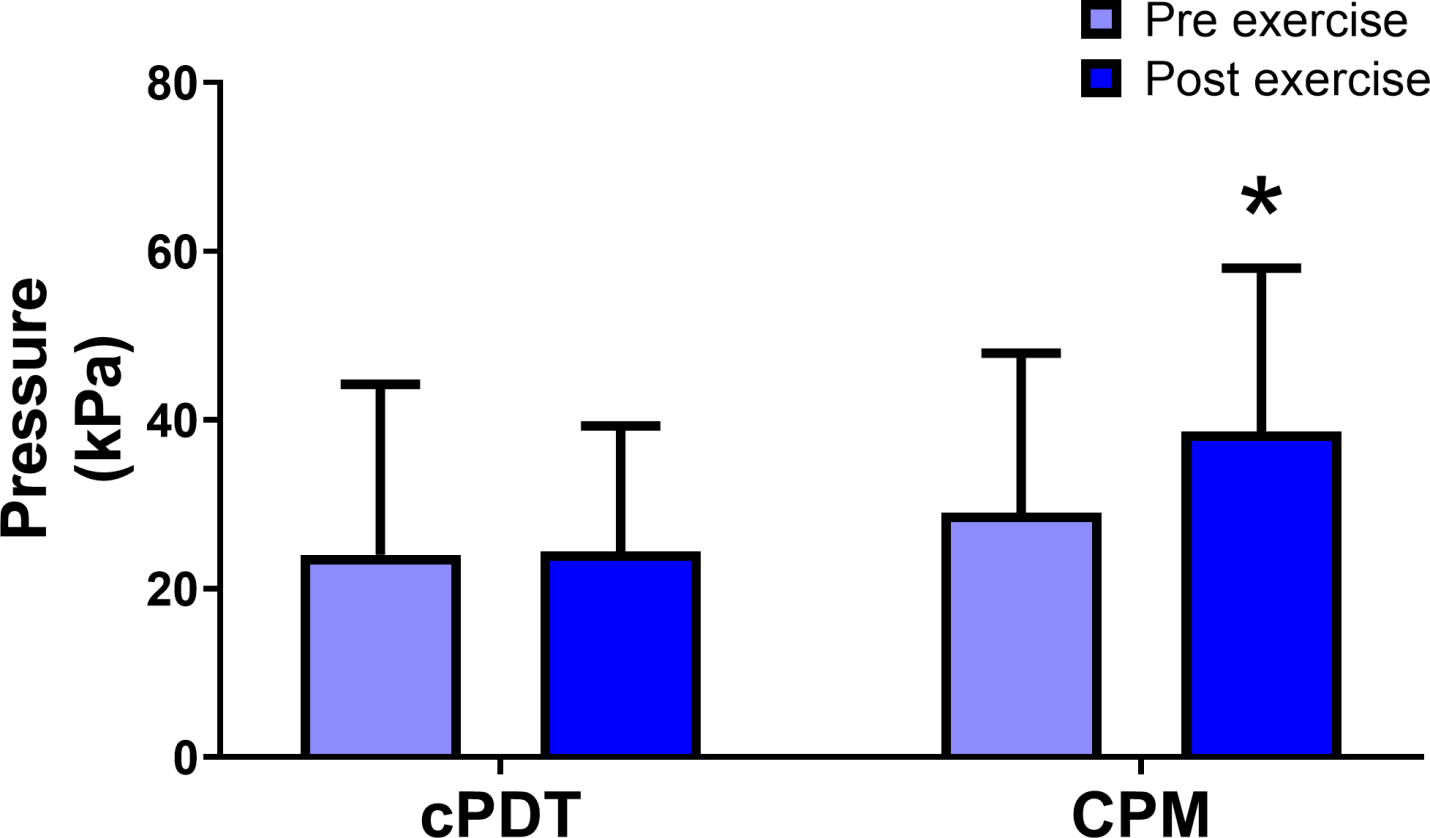
Pre‐ and post‐exercise cuff pressure detection threshold and conditioned pain modulation. Results of cuff pressure detection threshold (cPDT) and conditioned pain modulation (CPM) pre‐ and post‐exercise measured in kilopascal (kPa). Errors bars represent standard error of measurement (SEM). *indicates statistical significance.

**FIGURE 4:**
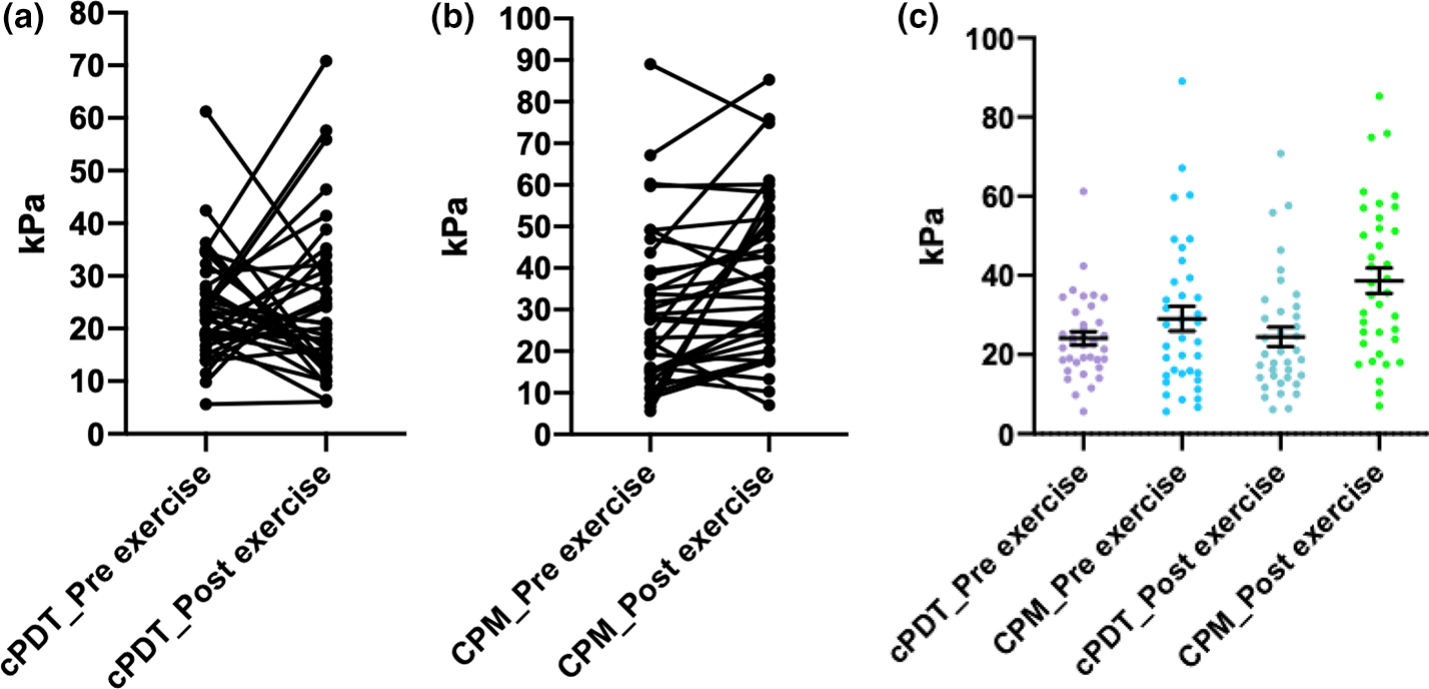
Individual participant data for pre‐ and post‐exercise cuff pressure detection threshold and conditioned pain modulation. Individual participant data of cuff pressure detection threshold (cPDT) and conditioned pain modulation (CPM) pre‐ and post‐exercise measured in kilopascal (kPa). Errors bars (yellow) represent standard error of measurement (SEM).

For the total group, cPDT measured using cuff algometry was 24 ± 10.2 and 24.4 ± 14.9 at baseline and follow‐up, respectively, with no change following exercise 0.3 ± 16.9 95% CI [0.6, 5.4], *p* = 0.44. No significant changes in PPTs prior to wall‐squat exercise, at either site or time point, were observed (*p* > 0.05) (Table 3). Similarly, paired sample t‐test showed no significant change in EIH 8 weeks after exercise compared to baseline EIH (*p* > 0.05) (Figures 3 and 4).

**TABLE 3:** Linear regression analysis of clinical pain

	Standardized β‐values	*p*
Model 1		
Worst pain		
Adjusted R^2^	33.8%	
cTSP	−0.214	0.201
PSE‐Q	−0.156	0.408
PCS	−0.137	0.498
Adherence	−0.07	0.517
CPM	−0.082	0.602
SPADI	−0.043	0.807
Pin prick	−0.014	0.933
PSQI	0.005	0.976
Model 2^a^		
cTSP	−0.240	0.0900
Worst pain	0.603	0.00

Abbreviations: CPM, Conditioned Pain Modulation; cTSP, (cuff) Temporal Summation of Pain; EIH, Exercise‐induced Hypoalgesia.; PCS, The Pain Catastrophizing Scale; PD, PainDETECT; PPT, Pressure Pain Threshold; PSE‐Q, Pain Self‐Efficacy Questionnaire; PSQI, Pittsburgh Sleep Quality Index; SPADI, Shoulder Pain and Disability Index.

^a^ Model 2 shows the best and final model out of 7 total models.

#### 3.3.2 Conditioned pain modulation

For the total group, CPM was 29 ± 18.9 and 38.6 ± 19.4 at baseline and follow‐up, respectively, with a significant change following exercise 9.5 ± 16.9 95% CI [3.9, 15.2], *p* < 0.001.

#### 3.3.3 Temporal summation of pinprick pain

For the total group, TSP measured using pinprick was 2.5 ± 2.0 and 2.2 ± 1.6 at baseline and follow‐up, respectively, with no change following exercise (0.21 ± 2.7 95% CI [−0.44, 1], *p* = 0.642).

#### 3.3.4 Temporal summation of cuff pain

For the total group, TSP measured using cuff algometry was 2.8 ± 1.6 and 3.0 ± 2.2 at baseline and follow‐up, respectively, with no change following exercise 3.2 ± 16.5 95% CI (−2.2, 8.7), *p* = 0.242.

### 3.4 Prediction of pain relief after the intervention

Several linear regression models were established to investigate if baseline parameters could predict the pain relief after the intervention. Model 1 included all the baseline parameters (QST, pain intensity and questionnaires) and demonstrated a predictive value (adjusted R^2^) of 23.2% in the prediction of absolute change in clinical pain and a predictive value (adjusted R^2^) of 21.7% for percentage change in clinical pain (Table 4). Model 2 applied backward elimination to model 1 to identify independent parameters and found that baseline pain intensity and cuff TSP predicted absolute change in clinical pain with a predictive value (adjusted R^2^) of 33.8% and percentage change in clinical pain with a predictive value (adjusted R^2^) of 29.4%, indicating that baseline low pain intensity and high TSP scores were associated with a limited pain relief to the intervention with pre‐treatment pain as the only independent parameter.

**TABLE 4:** Linear regression analysis

	Standardized β‐values	*p*
Model 1		
Worst pain		
Adjusted R2	33.8%	
cTSP	‐0.214	0.201
PSE‐Q	‐0.156	0.408
PCS	‐0.137	0.498
CPM	‐0.082	0.602
SPADI	‐0.043	0.807
Pin prick	‐0.014	0.933
PSQI	0.005	0.976
Model 2^a^		
cTSP	‐0.240	0.0900
Worst pain	0.603	0.00

^a^ Model 2 shows the best and final model out of 7 total models.

## 4 DISCUSSION

This exploratory study in patients with unilateral subacromial pain syndrome was designed to assess if 8‐weeks of a simple home‐based shoulder exercise could modulate QST and if demographics, TSP, CPM and EIH could predict the pain relief to 8‐weeks of exercise. The exercise intervention significantly decreased clinical pain parameters, improved sleep quality, reduced the PainDETECT scores and increased CPM. Higher levels of pre‐treatment TSP (high degree of sensitisation) and lower levels of pre‐treatment clinical pain intensity predicted the least effects on clinical pain by the intervention.

### 4.1 Effect of prolonged exercise

Exercise for SAPS have previously shown to be effective with small to moderate effect sizes and is recommended as first line of care (Babatunde et al., 2021; Rohit et al., 2015). Despite this, recent larger trials have questioned the effectiveness of exercise, with one study showing that 24%–27% of patients with SAPS experienced much improvement or full recovery and 50% experienced acceptable improvements in pain after 4 months of exercise (Clausen et al., 2021). A large multicentre trial failed to show any difference between supervised exercise compared to best practice advice with and without injection of corticosteroids and reported that best practice advice and injection was the most cost‐effective approach after 12 months (Hopewell et al., 2021). The current study found a significant reduction in clinical pain intensity, but this did not meet the requirement for MCID (Cook, 2013; Michener et al., 2011; Werner et al., 2016). A recent meta‐analysis showed a positive correlation between longer duration of an exercise intervention and the pain reduction in other chronic musculoskeletal pain conditions (Clausen et al., 2021; Hopewell et al., 2021; Polaski et al., 2019) and it its plausible, that the length of the interventions is important for the effect. Another reason for the lack of clinically relevant effect might be explained in the magnitude of load that were encountered in this study. In a study from Holmgren et al., a large dose of exercise was found to be more effective compared to a low dose of exercise in terms of improving pain and function (Holmgren et al., 2014). The dose–response relationship was further analysed in Clausen et al. comparing a large added dose to current conservative care and concluded that a larger dose did not result in improved outcomes (i.e., SPADI‐score) independent of post‐hoc analysis (Clausen et al., 2021). The effectiveness of prolonged periods of exercise is therefore still debatable and future research should investigate if exercise can be further optimized in conjunction with other modalities including patient education (Chester et al., 2018; Rathleff et al., 2018), psychological treatment or shared decision‐making (Tousignant‐Laflamme et al., 2017).

### 4.2 Modulation of mechanistic pain biomarkers after exercise

Several studies are suggestive that specific QST parameters can be modulated by exercise in healthy individuals including PPT (Hosseinzadeh et al., 2013), TSP (Vaegter et al., 2015) and EIH (Hansen et al., 2020a). Other studies have shown that PPT (Lyng et al., 2021) and CPM (Graven‐Nielsen et al., 2012) can be increased by exercise, manual therapy, and surgery in patients with musculoskeletal pain conditions. A recent study showed that 5‐weeks of eccentric training increased CPM, but not TSP in females with neck pain (Heredia‐Rizo et al., 2018). Heredia‐Rizo et al. 2019 implemented an advanced exercise programme using a custom‐built dynamic shoulder dynamometer, which is not easily implemented in clinical practice, opposed to our study. Another study showed that high intensity exercise (i.e., in adjunction to neuromuscular exercise and education) was shown to impose significant increase in PPTs and PTT, but not TSP or CPM in patients with knee osteoarthritis compared to neuromuscular exercise and education alone (Holm et al., 2021). Similar findings were support by Hansen et al. who showed that the EIH and clinical pain improvement were associated (Hansen et al., 2020b). The current study demonstrated an improvement (increasing the inhibitory effect) in CPM over the course of 8‐weeks and therefore adds to the literature that exercise therapy might improve central pain mechanisms. CPM can be assessed using a range of different combinations and studies have found that some exercise intervention might influence CPM whereas other might not influence CPM (Imai et al., 2016). Heredia‐Rizo et al. 2019 used a pressure‐based CPM test to assess patients with neck/shoulder pain and found exercise to increase CPM (Lyng et al., 2021; Heredia‐Rizo et al., 2018; Heredia‐Rizo et al., 2020). Neziri et al., 2013 argued that pressure stimuli were better at decimating healthy individuals from patients with chronic neck pain and therefore, it could be speculated that pressure stimuli might be more relevant for patients with musculoskeletal pain (including should pain) and therefore the pressure CPM test might be better suited for these patients (Neziri et al., 2013). To our knowledge, there are no studies which have applied multimodal CPM protocols to investigate which combination of modalities can be influenced by prolonged exercise, but future studies could investigate this. The variability of QST parameters is large and reliability of CPM has recently been discussed (Kennedy et al., 2016). As mentioned, CPM can be assessed using a range of different tools and it seems evident that the different CPM assessment will provide different results, which does complicate generalizability of study results (Imai et al., 2016; Vaegter et al., 2018). Older studies have argued that patients with chronic pain demonstrate an impairment in CPM when compared to healthy pain‐free individuals, but recent studies have demonstrated both impairments and facilitation of CPM in both patients with chronic pain and healthy pain‐free individuals (Arendt‐Nielsen et al., 2018; Izumi et al., 2022). The variability of C can partially be explained by the many factors that seems to impact the CPM methodology and for example, sleep deprivation and levels of pain catastrophizing have been shown to modulate CPM results (Staffe et al., 2019; Christensen et al., 2020). Increasing evidence suggest that the variability in CPM can potentially be utilized to predict treatment outcomes and the current study adds to this increasing evidence (Petersen et al., 2020). It is however also evident, that the predictive strength of CPM is low‐to‐moderate, which is in line the findings from the current study.

### 4.3 Modulation of sleep after exercise

Sleep deprivation is known to negatively affect QST parameters (Staffe et al., 2019), be associated with worsening of clinical pain (Campbell et al., 2015) and to predict the development of chronic pain after surgery (McBeth et al., 2015; Mork & Nilsen, 2012). Sleep deprivation might be linked to increase in sensitization of pain mechanisms possible via low‐grade inflammation (Tousignant‐Laflamme et al., 2017) (Schaible, 2014). Exercise can improve sleep quality, and promote an anti‐inflammatory response, which potentially could lead to improvements in QST parameters (Reid et al., 2010; Lü et al., 2017; Leung et al., 2016; Mosser & Edwards, 2008). However, the various study varies substantially in the modality of the exercise. The current study did not find improvements in both sleep quality and increased CPM, most likely due to the low intensity of the home exercise program.

### 4.4 Predicting response to exercise by mechanistic pain biomarkers

Increasing evidence suggest that pre‐treatment QST assessments can predict the treatment responses to standard pain treatments (Petersen et al., 2020), some types of surgery (Petersen et al., 2016; Petersen et al., 2015; Wilder‐Smith et al., 2010), weak analgesics (Petersen et al., 2019b; Petersen et al., 2019c; Edwards et al., 2016) and exercise therapy (Hansen et al., 2020b; O'Leary et al., 2018) in various pain conditions. Studies have demonstrated that combining QST modalities with other relevant clinical pain outcomes may increase the value of the prediction models (Yarnitsky et al., 2012; Larsen et al., 2021). Cognitive factors such as depression, anxiety and pain catastrophizing are found as predictive for treatments outcomes (Edwards et al., 2011) and it seems that these factors are also worsened by poor quality of sleep (Larsen et al., 2021). The current study is the first to find that TSP is predictive of the lack of pain reduction to an exercise protocol in patients with unilateral SAPS, which is found in other painful musculoskeletal conditions (Petersen et al., 2020). The current study was unable to demonstrate find that poor quality of sleep was a predictor for the treatment response and larger studies with a focus on variety of cognitive are recommended to further explore this area.

### 4.5 Limitations

This study was conducted as an explorative study investigating a cohort of participants diagnosed with unilateral SAPS. The main limitation of this explorative study was the lack of control groups that hampers the validity of the findings from this study, hence the effectiveness should interpretated with caution. The use of parallel or sequential CPM designs have been discussed (Yarnitsky et al., 2015). In a recent study from Reezigt et al. the differences between CPM design were investigated and the authors concluded that only minimal to no differences were present (Reezigt et al., 2021). Research have shown that CPM varies in both patients with chronic pain and in healthy pain‐free individuals (Kennedy et al., 2016; Izumi et al., 2022). Participants were equipped with various elastic resistance bands for home training to accommodate both increase in strength and general level of strength around the shoulder. While this enabled the individual to adapt the resistance to their needs, free weights are often used as a fitting alternative to individualized training with the aim of increasing muscular strength. Addressing this, a recent meta‐analysis showed no superiority between either methods or it is therefore unlikely that the given method had a real impact on the intervention (Lopes et al., 2019). No data were available to prove if patients complied with the instructions given and if they followed the instructed exercise schedule.

## 5 CONCLUSION

This exploratory study demonstrated that progressive home‐based shoulder abduction exercise over a period of 8‐weeks resulted in significant reductions in pain, but not clinical meaningful, and led to an increased CPM and sleep quality in patients diagnosed with unilateral SAPS. Additionally, TSP and clinical pain on NRS assessed before initiation of the exercise program predicted pain alleviation assessed as the absolute and percental response to therapy with a prediction value of 29.4–33.8%. Future confirmatory studies are needed to validate these findings.

## FUNDING INFORMATION

The project is financed by funds from The Aalborg University Talent Management Programme (j.no. 771126) and Danish Society of Sports Physical Therapy (DSSF). No of the funders were included in any steps of the design, data acquisition, analysis, or interpretation.

## CONFLICTS OF INTEREST

None of the authors have any conflicts of interest to declare.

## Supporting information


Appendix S1
