# Supplementary material for: The influence of exercise on clinical pain and pain mechanisms in patients with subacromial pain syndrome
Source: Eur J Pain. 2022 Jul 27;26(9):1882–95. doi: 10.1002/ejp.2010 (PMC9545950; doi:10.1002/ejp.2010)
Supplement: Supplementary file 1 — Appendix S1 [file EJP-26-1882-s001.docx]

**Supplementary material**

**SUPPLEMENTARY MATERIAL: DEMOGRAPHICS INCLUSION**

**TABLE 1: TIDieR CHECKLIST**

| **Name** | **Why?** | **What?** | **Who?** | **How?** | **Where?** | **When and how much?** | **Tailoring** | **Modification** | **How well?** |
| --- | --- | --- | --- | --- | --- | --- | --- | --- | --- |
| Shoulder scaption exercise (abduction). | It has never been investigated which potential effects, this simple exercise program has on quantitative sensory testing measurements and which factors could influence the outcome. | KDL will deliver the information regarding the intervention. The exercise is a shoulder abduction.   The exercise is based on previous studies comparing usual care to a simple home- based exercise for shoulder pain. | The exercise will be prescribed by the KDL. | Face to face instructions, home based during the weeks afterwards | Home-based, individually | The home- based exercise will be performed twice a day, every other day, for 3 sets of 15 repetitions.   The time- under- tension would be three seconds concentric and eccentric abduction and no rest in top or bottom position.   The participant will ideally participate in 64 training session during the 8 weeks of the intervention.  The training load will be 15RM, gradually progressing. | All participants will be provided with several therapeutic bands with various tension strength to match progression of pain or progression of strength. | Each participant will be instructed to execute the exercise with no compensatory movements not related to the shoulder.   Each participant will be asked to pick the load that they will be able to perform for the prescribed dose.   Intolerable pain reported by the patient will be reported and the participant will be instructed to reduce the load for the next 3 days. | Each participant will be asked to write down number of sessions/repetitions/sets completed in a diary. Pain experienced during the exercise- session will be reported by the participant, rated on an NRS-scale. |
